# Bilateral simultaneous anterior ischemic optic neuropathy, an extrahepatic manifestation of hepatitis C cured with direct acting antivirals

**DOI:** 10.3205/oc000042

**Published:** 2016-04-04

**Authors:** Sylvie Prud’homme, Frederik Nevens, Ingele Casteels

**Affiliations:** 1 Division of Ophthalmology, University Hospitals KU Leuven, Leuven, Belgium; 2 Division Liver and Biliopancreatic Disorders, University Hospitals KU Leuven, Leuven, Belgium

**Keywords:** non arteritic ischemic optic neuropathy, hepatitis C, sofosbuvir, daclatasvir

## Abstract

We report a patient with a bilateral optic anterior ischemic neuropathy as an extrahepatic complication of a chronic hepatitis C (HCV) infection. The patient presented with a bilateral visual acuity loss and bilateral optic disc oedema. The optic neuropathy was associated with a sudden increase in the viral HCV load after a recent liver transplantation. The stop of the calcineurin inhibitor had no effect on the course of the optic neuropathy. Visual improvement and normalization of HCV viraemia occurred after treatment with sofosbuvir and daclatasvir, which are direct acting antivirals.

## Introduction

Chronic hepatitis C virus (HCV) infection is a global epidemic affecting almost 185 million people worldwide, with an estimated 3–4 million new infections every year [1]. HCV infection can lead to the development of liver cirrhosis, hepatocellular carcinoma and liver failure [2]. HCV exhibits an extraordinarily high degree of genetic diversity. HCV genotype 1 is the most prevalent worldwide [3]. Several extrahepatic manifestations of HCV have been described such as cryoglobulinemia induced vasculitis [4], [5] and a possible relationship with non-arteritic anterior ischemic neuropathy (NAION) [6].

## Case report

**Figure 1:**
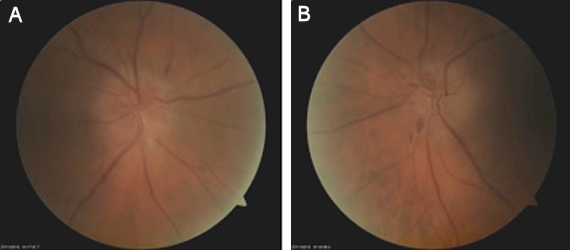
Papillary oedema at the begining in A) the right eye and B) the left eye

**Figure 2:**
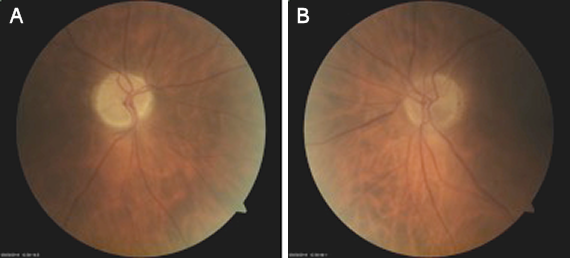
Atrophic optic nerve at the end of treatment in A) the right eye and B) the left eye

A 68-year-old man was referred to our hospital with a deteriorated general condition due to chronic hepatitis C genotype 1b. In this advanced stage of disease, treatment with pegylated interferon, ribavirin and a direct acting antiviral (DAA) was not indicated. Progressive cirrhosis necessitated a liver transplant 2 years later. The pre-transplant ophtalmological examination was performed 6 months before the actual liver transplantation and was completely normal. Following the transplantation, treatment with triple immunosuppression with tacrolimus, mycophenolatic mofetil and prednisolone was initiated. Tacrolimus was replaced by cyclosporine because of confusion. Three months later, the liver function had normalized with therapeutic blood levels of cyclosporine around 75 µg/l, the viral load at that moment was 130,499 IU/ml. Four months after the liver transplantation, the patient developed an acute visual loss in both eyes. Best-corrected visual acuity was 0.6 in the right eye and 0.12 in the left eye. There was a relative afferent pupillary defect in the right eye. On fundoscopy, bilateral optic disc oedema with haemorrhages at the disc margin was seen (Figure 1 (Fig. 1)). Treatment with cyclosporine A was interrupted and the dose of prednisolone was increased to 24 mg in combination with mycoplenolatic mofetil. One month later his vision was at 0.4 in the right eye and 0.25 in the left eye. The papillary oedema gradually subsided and the optic nerves became pale. Goldmann perimetry showed normal peripheral limits and central visual field testing showed a superior nasal scotoma in the right eye and an inferior nasal scotoma in the left eye. Brain MRI (magnetic resonance imaging) was within normal limits. A blood sample could not detect any infectious causes of an optic neuropathy, but an HCV viral load was >5 million IU/ml was measured. Patient was closely monitored, but after one month there was no visual improvement and optic pallor was progressive bilaterally; the dosage of prednisolone was increased to 2 x 32 mg. Six months after transplantation liver function tests deteriorated and the patient developed a histologically proven fibrotic cholestatic hepatitis. Antiviral therapy with peginterferon alpha 2a and ribavirin was given for one month. During that month BCVA slightly decreased and visual fields deteriorated. After this month, seven months after the liver transplant, therapy was switched to ribavirin and sofosbuvir. Afterwards visual acuity stabilized and Goldmann perimetry showed a scotoma inferior and nasal in the right eye and a narrowing of the peripheral field in the left eye. Central visual field examination showed an increase of the inferior nasal scotoma in both eyes. Ten months after the transplant, therapy was switched to daclatasvir and sofosbuvir because of the availability of DAA (direct acting antiviral) in Belgium. Prednisolone was progressively tapered and cyclosporine was reintroduced. The viral load dropped to 373 IU/ml. Over the following months, vision improved slightly to 0.7 in the right eye and to 0.5 in the left eye. The peripheral and central visual fields improved. The optic nerves had become atrophic (Figure 2 (Fig. 2)). Liver function test normalized and the viral load became undetectable. Three months after the start of daclatasvir and sofosbuvir the patient was a sustained responder and the liver test was completely normal.

## Discussion

We report a patient with bilateral NAION in a patient with a chronic hepatitis C infection. We assume a relationship between the NAION and HCV, since he had a high viral load (>5 million IU/ml) during the NAION. Treatment with direct acting antivirals resulted in improvement of visual acuity and of the fibrotic cholestatic hepatitis. We do not believe this is a cyclosporin-induced NAION, since stopping this medication had no effect on the visual acuity.

Fodo et al. reported a case of a consecutive bilateral NAION presumably caused by hepatitis C virus type 1b [6]. The patient developed an NAION in both eyes with a time interval of five months. Viral load was very high when the contralateral NAION developed, as it was in our patient. Brain MRI was found to be normal. The patient was treated with pegylated interferon and ribavirin. No further deterioration occurred when antiviral therapy was started. 

The association between a bilateral optic neuropathy and a hepatitis C infection has been described before by Siddiqui et al. [7]. Optic neuropathy emerged 2 weeks after an acute hepatitis C infection. Vision recovered completely after treatment with steroids. Optic neuropathy in patients with hepatitis A and B [8], [9], [10] has also been described 2 months after an episode of acute hepatitis. In most patients treatment with steroids was successful. 

Optic neuropathy following the use of cyclosporine has been linked in patients with a T-cell lymphoma [11]. Here, a marked improvement of the vision and the visual field was noted once cyclosporine was stopped. 

Until recently treatment for chronic hepatitis C infection consisted of pegylated interferon alpha and ribavirin. One of the most frequent complications is an interferon-associated retinopathy [12], [13]. This retinopathy occurs in 30–60% of cases. Since interferon was only given for a short period of time some months after the start of the NAION, we don’t believe this medication had an effect on the course of the visual symptoms.

At present, DAAs that do not require pegylated interferon provide new opportunities for treatment of HCV. They target specific steps within the HCV life cycle. A combination of these DAAs without pegylated interferon with a treatment duration of 12–24 weeks offers a sustained response rate in genotype 1b patients of >90% without any significant adverse events even after liver transplantation [14], [15]. In our case we show that these drugs are also successful for the HCV induced fibrotic cholestatic hepatitis seen after liver transplantation, which is a life-threatening condition without antiviral treatment. 

## Conclusions

In conclusion, we report a case of bilateral optic NAION witch is presumably an extrahepatic complication of a chronic hepatitis C infection. One case has been reported in literature [6]. In our patient, we can assume that a reactivation of a chronic hepatitis C after liver transplantation was the triggering factor for the NAION, whereas in the other case it was an HCV infection that had not been treated before [6]. It is less likely that the cyclosporine was responsible for this, since ceasing them had no effect on the course. 

In our patient the viral load was very high (>5 million IU/ml) during the NAION; viral load was not this high before. Vision stabilised and even improved after starting DAA. This is also the moment when the viral load started to decline and eventually became undetectable. In our case we see correlation between the viral load and the start of the visual deterioration of the patient. This is the second case report where a correlation between the development of an NAION and HCV can be assumed. Our case is the first case where you can see improvement in vision when the viral load dropped after a DAA was started. 

Many other patients with HCV receive pegylated interferon treatment, which is responsible for a lot of visual side effects, but this is not the case in our patient since he did not receive this therapy.

This case also illustrates again the efficacy of the combination of DAAs in the eradication of a chronic hepatitis C infection in liver transplant patients suffering from fibrotic cholestatic hepatitis.

In conclusion this case seems to show that NAION can be caused by the virus itself and not only as a complication of the used therapy. In this case, targeting the cause of the NAION prevented further deterioration. 

## Notes

### Competing interests

The authors declare that they have no competing interests.
